# Positive Selection Targeted Primate Genes that Encode Transposable Element Repressors

**DOI:** 10.1093/gbe/evag059

**Published:** 2026-03-05

**Authors:** Rachele Cagliani, Diego Forni, Alessandra Mozzi, Roudin Sarama, Uberto Pozzoli, Matteo Fumagalli, Manuela Sironi

**Affiliations:** Scientific Institute IRCCS E. MEDEA, Computational Biology Unit, Bosisio Parini 23842, Italy; Scientific Institute IRCCS E. MEDEA, Computational Biology Unit, Bosisio Parini 23842, Italy; Scientific Institute IRCCS E. MEDEA, Computational Biology Unit, Bosisio Parini 23842, Italy; School of Biological and Behavioural Sciences, Queen Mary University of London, London, UK; Scientific Institute IRCCS E. MEDEA, Computational Biology Unit, Bosisio Parini 23842, Italy; School of Biological and Behavioural Sciences, Queen Mary University of London, London, UK; School of Medicine and Surgery, University of Milano-Bicocca, Monza, MB 20900, Italy; Fondazione IRCCS San Gerardo dei Tintori, Monza, MB 20900, Italy

**Keywords:** transposable element control genes, positive selection, intrinsically disordered regions, PIWIL proteins

## Abstract

Transposable element (TE) mobilization poses a significant fitness challenge to host genomes. Consequently, a variety of systems have emerged to silence TE activity. Just like TEs, such systems are widespread and their evolution is expected to be shaped by intra-genomic conflicts. To test this hypothesis, we performed an evolutionary analysis of TE control systems across different timescales. We show that a substantial fraction of TE control genes were targets of positive selection during primate evolution, with several proteins of the piRNA-pathway showing a considerable number of positively selected sites. In these proteins, selection was strongest in intrinsically disordered regions (IDRs), particularly those with low conformational entropy. We suggest that positive selection modulates IDR properties by introducing changes in sequence patterns and ensemble features properties, which are in turn related to function. Analysis of genetic data from 54 human populations detected several signals of strong positive selection at TE control genes. In line with findings in primates, we identified as selection targets three genes (*TEX15*, *GTSF1*, and *GTSF1L*) that participate in the piRNA pathway. Additionally, 4 of the 13 genes with strong evidence of positive selection encode components of the NuRD complex, which plays central functions not only related to TE control but also to the maintenance of genome integrity and cell cycle control. Our data provide insight into the evolution of TE control systems in primates and human populations. Whereas the signatures we detected are consistent with a genomic conflict between TEs and their repressors, additional pressures may drive the evolution of the genes we analyzed.

Significance Transposable element (TE) control systems play an essential function in the maintenance of genome integrity. We analyzed the evolutionary history of genes that silence transposable elements across different timescales. Our working hypothesis was that the evolution of such systems has been dynamic, as a result of genetic conflicts with TEs. Indeed, we found that genes that control TE activity were common targets of positive selection in primates and during the more recent evolution of human populations, suggesting ongoing pressure exerted by extant mobile elements. Our data sheds light on the diversity and breadth of intra-genomic conflicts, and opens new perspectives to identify variants that might modulate TE mobilization in humans.

## Introduction

Transposable elements (TEs) are mobile genetic units that are able to move and amplify within host cells. TEs have successfully populated eukaryotic and prokaryotic genomes, and the typical mammal has ∼45% of its genome derived from TEs (Venter et al. 2001; De Koning et al. 2011; Haudiquet et al. 2022; Osmanski et al. 2023). TEs are highly diverse, but can be classified into two major groups depending on the mobilization mechanism (Finnegan 1989; Wicker et al. 2007). Class I elements, also known as retrotransposons, propagate through an RNA intermediate. These include short interspersed nuclear elements (SINEs), long interspersed nuclear elements (LINEs), and long terminal repeat (LTR) retrotransposons (Wicker et al. 2007). Class II TEs, also known as DNA transposons, use a DNA intermediate to propagate (Kapitonov and Jurka 2006; Thomas and Pritham 2015). In mammalian genomes, the large majority of TEs belong to class I (Wells and Feschotte 2020).

Although increasing evidence indicates that they significantly contribute to adaptive genome evolution, TEs behave as selfish elements, and their replication at the genomic level can be detrimental (Doolittle and Sapienza 1980; Jangam et al. 2017; Bourque et al. 2018; Joly-Lopez and Bureau 2018; Schrader and Schmitz 2019; Almojil et al. 2021; Almeida et al. 2022). TEs can be mutagenic, either directly (e.g. by insertional disruption of coding or regulatory sequences) or indirectly (e.g. by mediating chromosomal rearrangements), and TE insertions can affect the regulatory environment of nearby genes (Almeida et al. 2022). For these reasons, cellular organisms have evolved several mechanisms to suppress TE activity and to limit its propagation. This is particularly true in the germline, where TE mobilization can lead to vertical transmission of new genomic copies. In mammals, a major TE control pathway hinges on P-element induced Wimpy testis (PIWI)-interacting RNAs (piRNAs). piRNAs form effector complexes with Piwi proteins (Piwis), the latter a germline-specific class of Argonaute proteins (Agos). These complexes guide recognition and silencing of TEs at the transcriptional or post-transcriptional level (Iwasaki et al. 2015; Ozata et al. 2019). For transcriptional silencing, complexes of piRNAs and Piwi use specific interactors (e.g. SPOCD1 and TEX15 in mammals) to promote the recruitment of DNA methyltransferases (DNMTs) and chromatin remodeling complexes to generate a repressive chromatin environment (Almeida et al. 2022). In the case of post-transcriptional control, piRNAs complexed with Piwi, together with co-factors, promote the degradation of TE transcripts in the cytoplasm (Almeida et al. 2022; Arif et al. 2022). One extremely relevant cofactor is GTSF1 (Gametocyte-specific factor 1), which binds the ternary complex of piRNA•PIWI•target to potentiate the cleavage activities of Piwi proteins (Arif et al. 2022). In mammals, the piRNA/Piwi pathway is particularly important in the germline. As a consequence, mutations in several components of the pathway result in the sterility of one or both sexes (Almeida et al. 2022).

Another mechanism of TE control is based on Krüppel-associated box (KRAB) domain-containing zinc finger proteins (KZFPs), which are encoded by an expanded gene family in vertebrates (Thomas and Schneider 2011). Through the KAP1/TRIM28 interactor, KZFPs recruit heterochromatin silencing factors, including DNMTs, heterochromatin protein 1 (CBX1/CBX3), SETDB1, and the NuRD complex (nucleosome remodeling and deacetylation complex). Moreover, additional systems exploit different TE features for recognition and silencing. For instance, the HUSH (human silencing hub) complex (which comprises TASOR, MPHOSPH8, and PPHLN1), together with its effectors (MORC2 and SETDB1), identifies DNA fragments with high adenine content in the sense strand and epigenetically represses them (Seczynska and Lehner 2023). Other mechanisms of TE suppression are instead based on RNA modifications, including N6-methyladenosine, 3′ uridylation, and 5-hydroxymethylcytosine (Almeida et al. 2022).

Because several proteins involved in TE control bind nucleic acids, they are expected to be enriched in intrinsically disordered regions (IDRs) (Liu et al. 2006; Zhao et al. 2021). IDRs do not adopt a stable three-dimensional structure under physiological conditions, but rather exist in conformational ensembles of energetically accessible, rapidly interconverting structures (Holehouse and Kragelund 2024). IDR amino acid composition, as well as the clustering and patterning of charged, polar, and hydrophobic residues, determine key properties of the conformational ensemble and, consequently, modulate functional properties (Holehouse and Kragelund 2024). IDRs are abundant in mammalian proteomes and, as a consequence of their limited structural constraints and tolerance to change, they are generally considered to evolve at a faster rate compared to structured regions (Holehouse and Kragelund 2024). This might be relevant in the context of TE control proteins, as the antagonistic effect exerted by cellular pathways against TE propagation is expected to result in intra-genomic conflicts (Hurst et al. 1996; Werren 2011). Indeed, TE propagation depends on evading cellular control systems and replication in the germline, while hosts need to silence TEs to ensure genome integrity and fertility. In line with these concepts, it was previously shown that KZFPs diverged and amplified in vertebrate lineages in response to the emergence of novel retroelements (Thomas and Schneider 2011; Jacobs et al. 2014) and de novo DNA methyltransferases show dynamic evolution in primates and rodents (Molaro et al. 2020). In *Drosophila*, a significant subset of piRNA pathway genes evolved under positive selection, and the same holds true for teleost fishes (Obbard et al. 2009; Lee and Langley 2012; Simkin et al. 2013; Yi et al. 2014; Parhad and Theurkauf 2019). Recently, signals of adaptive evolution were also reported in the HUSH complex effector MORC2 in primates (Lasserre et al. 2023).

Recent TE activity has been documented in the human genome and about 100 evolutionarily young LINE1 elements are still mobile in our genomes (Osmanski et al. 2023). These autonomous LINE1s provide the enzymatic machinery for their own integration, as well as for the insertion of the nonautonomous SINEs (*Alu* and SVA). Recent estimates indicated a retrotransposition rate for LINE1, Alu, and SVA ranging from one in 40 to one in 60 live births, depending on the element (Feusier et al. 2019). These new insertions have the potential to cause human genetic diseases, and elevated LINE1 expression and retrotransposition have been associated with many types of human cancers, as well as with male sterility (Hancks and Kazazian 2016; Yang and Wang 2016; Mendez-Dorantes and Burns 2023). Thus, TEs are likely to have exerted a selective pressure not only in the distant past but also during the more recent evolutionary history of human populations. A continuum in selective pressure acting on different timescales might therefore be expected, although distinct waves of TE expansion may exert pressure on distinct genes. In this study, we integrated molecular evolution analysis, population genetics, and structural biology to delve into the evolutionary history of TE control systems in primates and in human populations.

## Results

### Positive Selection Drives the Evolution of Several TE Control Genes

**Fig. 1.**
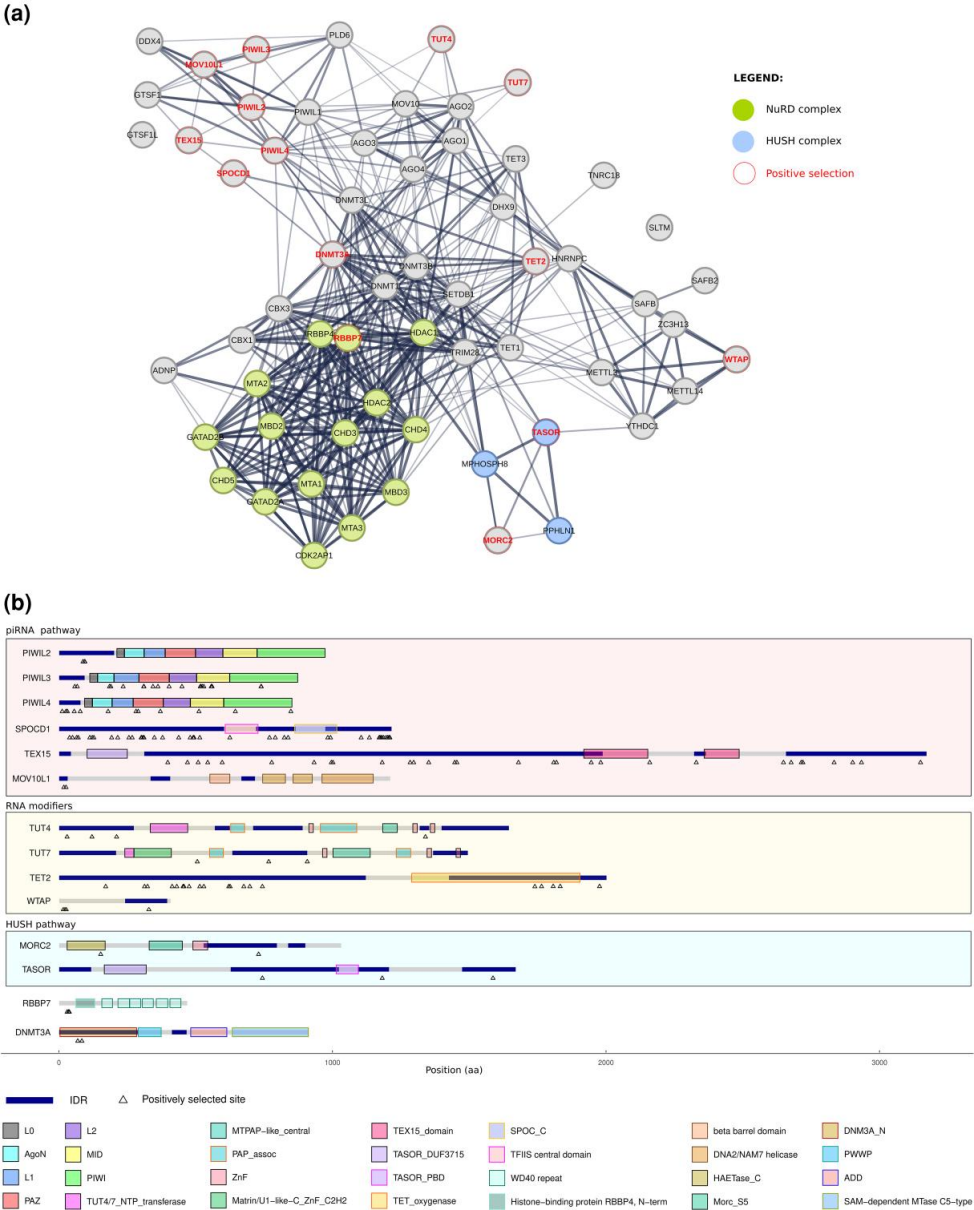
Evolution of TE control systems in primates. a) Protein-protein interaction network predicted for the 60 analyzed proteins. The network was generated using STRING (https://string-db.org/), which covers both physical interactions and functional associations between proteins. Proteins under positive selection in primates are colored in red. NuRD and HUSH complex components are shown in green and blue, respectively. b) Domain structures of the 14 proteins under positive selection in the primate phylogeny. Schematic domain structures of human proteins are drawn to scale. The protein domains were obtained from the InterPro website (https://www.ebi.ac.uk/interpro/) (Blum et al. 2025), with the exception of the Piwi proteins. The domains of PIWIL2 were obtained from Li et al. (2024), while the domains of PIWIL3 and PIWIL4 were defined by sequence homology from PIWIL2. IDRs identified by the Metapredict tool based on human proteins are shown in blue. Positively selected sites are shown below each domain structure as arrowheads.

We sought to test whether genes encoding products that suppress TE mobilization have been targets of positive selection during primate evolution. We thus assembled a list of sixty coding genes involved in TE control (Fig. 1a, Table S1). We first sought to determine whether the protein products of these genes are engaged in a genetic conflict with TEs. If this were the case, evidence of positive selection might be expected as a signature of fast evolution in response to TE challenge. We thus focused on primates and retrieved sequence information of the coding sequences from public databases, with the aim to analyze their evolutionary patterns (Table S1). To test the hypothesis that positive selection has been driving the evolution of TE control genes, we applied likelihood ratio tests (LRTs) implemented in the PAML suite (Yang 1997; Yang 2007). All genes were screened for recombination and split into different regions if necessary. The neutral models (M7 and M8a) were rejected in favor of the positive selection model (M8) for 14 genes, corresponding to a considerably high fraction of 23.3% (Fig. 1a and b, Table S2). The positively selected genes included three Piwi proteins and other molecules that participate in the piRNA pathway (SPOCD1 and TEX15), as well as components of the HUSH and NuRD complexes. In line with previous findings, we detected evidence of positive selection in MORC2 (Lasserre et al. 2023) and in the N-terminal region of DNMT3A (Molaro et al. 2020) (Fig. 1b).

**Fig. 2.**
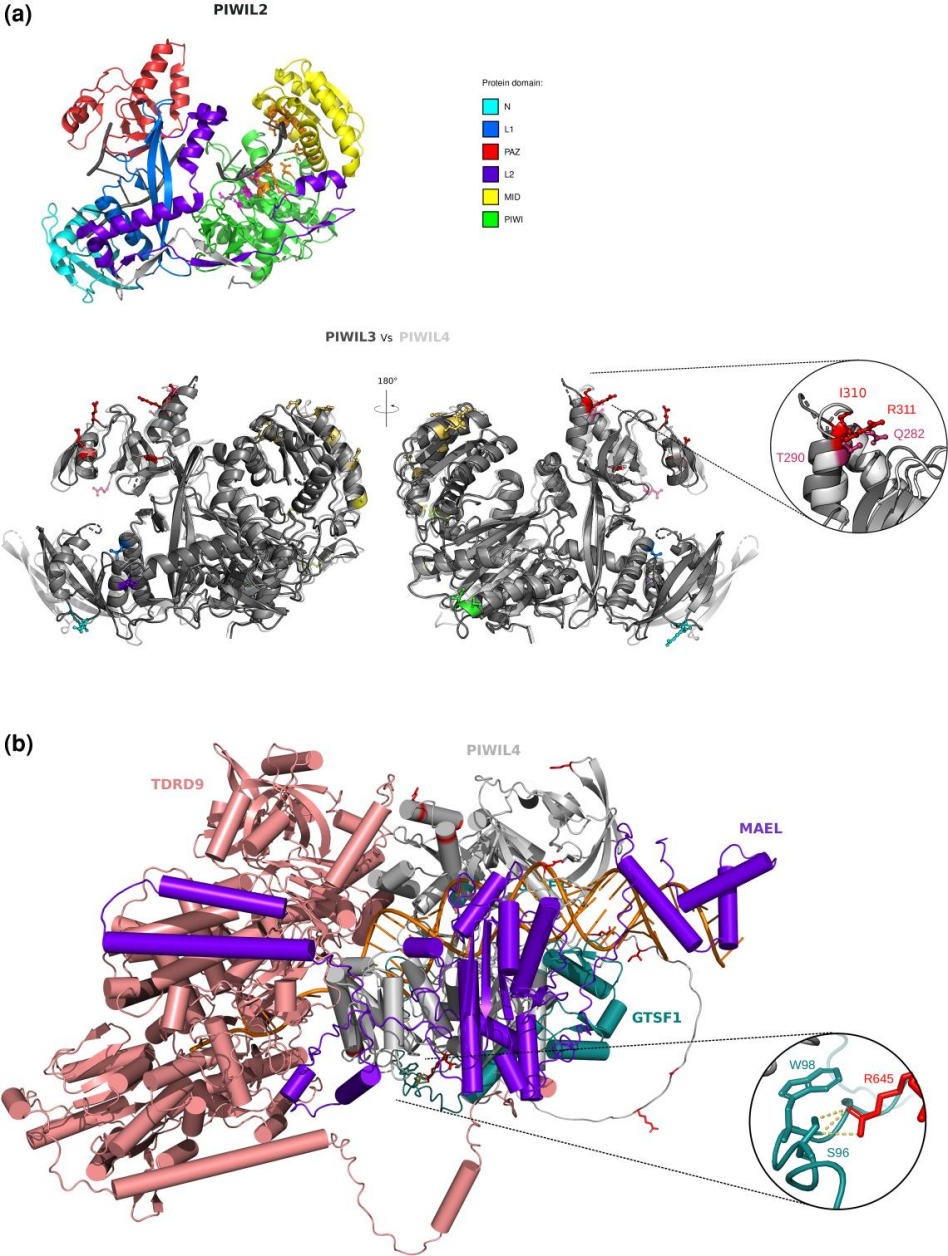
a) Cartoon representation of the 3D structure of human PIWIL2 in complex with piRNA (PDB ID: 7yfx). Protein domains are color-coded as reported in the legend. piRNA is in dark gray. Residues involved in piRNA recognition and the catalytic tetrad are represented as balls and sticks, in orange and in magenta, respectively. Below is reported the structural alignment of the human PIWIL3 (dark gray) AlphaFold model (AlphaFold DB: AF-Q7Z3Z3-F1-v4) with the human PIWIL4 (light gray) AlphaFold model (AlphaFold DB:AF-Q7Z3Z4-F1-v4) with the same spatial orientation. Positively selected sites are reported as ball and sticks, and color-coded according to the domain they map to (see legend). Positively selected sites in PIWIL3 (red) and PIWIL4 (magenta) falling in the same structural region are shown in the enlargement. b) Structural model of the human PIWIL4-GTSF1-MAEL-piRNA-target duplex-TDRD9 complex (prediction score = 0.726). PIWIL4 positively selected sites are in red. The positively selected residue (R645) that makes contact with GTSF1 is reported in the enlargement.

We were also interested in identifying the positively selected sites in these genes. Using a conservative approach (see methods), a total of 149 selected sites were identified (Table S2). The average fraction of selected sites per protein resulted equal to 0.88%, with the highest proportion being observed for SPOCD1 (3.45%). In general, components of the piRNA pathway were found to display a high number of selection signals (Fig. 1b). Among these, we detected three sites that were independent targets of selection in the PAZ (PIWI-Argonaute-Zwille) domains of the two paralogous genes PIWIL3 (I310 and R311, residue numbering according to the human protein) and PIWIL4 (Q282). These sites define the same structural patch in the 3D structures of the corresponding Piwi proteins (Fig. 2a), suggesting that changes in this region are functionally relevant and adaptive. Although a number of positively selected sites were found to be located in the MID and PAZ domains, none of them is involved in the binding of guide RNAs (Fig. 2a).

Given the recent resolution of PIWI* complex assemblies—which comprise a PIWI protein, a piRNA-target duplex, a GTSF protein and the Maelstrom protein—and that serves as molecular hub for downstream effectors, we reconstruct by structural modeling the human PIWIL2/3/4* complexes with TDRD9, which was found to be a general interactor of PIWI* complexes (De et al. 2025; Portell-Montserrat et al. 2025).

Only for PIWIL2* and PIWIL4*—TDRD9 we obtained models reaching confident prediction scores (>0.7). Whereas the positively selected residues detected in PIWIL2 lie in a disordered region that is not involved in interaction with assembly components, the R645 site of PIWIL4, which was identified as positively selected in the PIWI domain, maps to the interaction surface with GTSF1 (Fig. 2b and Fig. S1). In particular, in the model, the R645 residue makes a polar interaction with S96 and W98 of GTSF1. W98 is a conserved aromatic residue that, together with the other two conserved tryptophan residues, was previously suggested to be responsible for the interaction of GTSF1 with PIWI proteins (Arif et al. 2022).

These observations indicate that positive selection may have acted to modulate inter-protein interactions within the piRNA pathway.

### Positive Selection Signals in TE Control Genes are Enriched in Intrinsically Disordered Regions

The proteins encoded by these TE control genes have different domain architectures and, because several of them bind nucleic acids, they are expected to contain IDRs) (Liu et al. 2006; Zhao et al. 2021; Holehouse and Kragelund 2024). We thus sought to test this expectation and to determine whether, as shown for other proteins, IDRs represented preferential targets of positive selection (Cagliani et al. 2024a, 2024b). We thus predicted their location in the reference human proteome using Metapredict V2 (Emenecker et al. 2021, 2022). We found that IDRs are significantly more common in TE control proteins compared to the proteome average (IDR fraction in TE control proteins = 0.43, average IDR fraction in the human proteome = 0.29, Wilcoxon rank sum test *P*-value = 1.7 × 10^−5^), and some of them are very long. By analyzing the location of positively selected sites, we observed that a large number of them map to IDRs (Fig. 1b, Table S2). To determine whether this represents a statistically significant enrichment, we used a binomial test that accounts for the fraction of sites embedded in IDRs. We found that the proportion of sites in IDRs is indeed significantly higher than expected by chance (*P*-value = 1.40 × 10^−12^).

To test whether these results were robust to a different IDR prediction method, we obtained consensus disorder annotations provided by MobiDB (Piovesan et al. 2025) for all genes we analyzed. We found a very good correlation (Pearson's correlation coefficient = 0.855, *P* = 3.6 × 10^−18^) between the disordered fractions from Metapredict and MobiDB, with no evidence that either method over- or under-predicts the IDR fraction (Fig. S2). Consistently, the proportion of selected sites in IDRs obtained from MobiDB resulted to be significantly enriched (*P* = 5.92 × 10^−8^).

### Sequence Patterns and Ensemble Features Differ in IDRs Depending on the Selective Regime

**Fig. 3.**
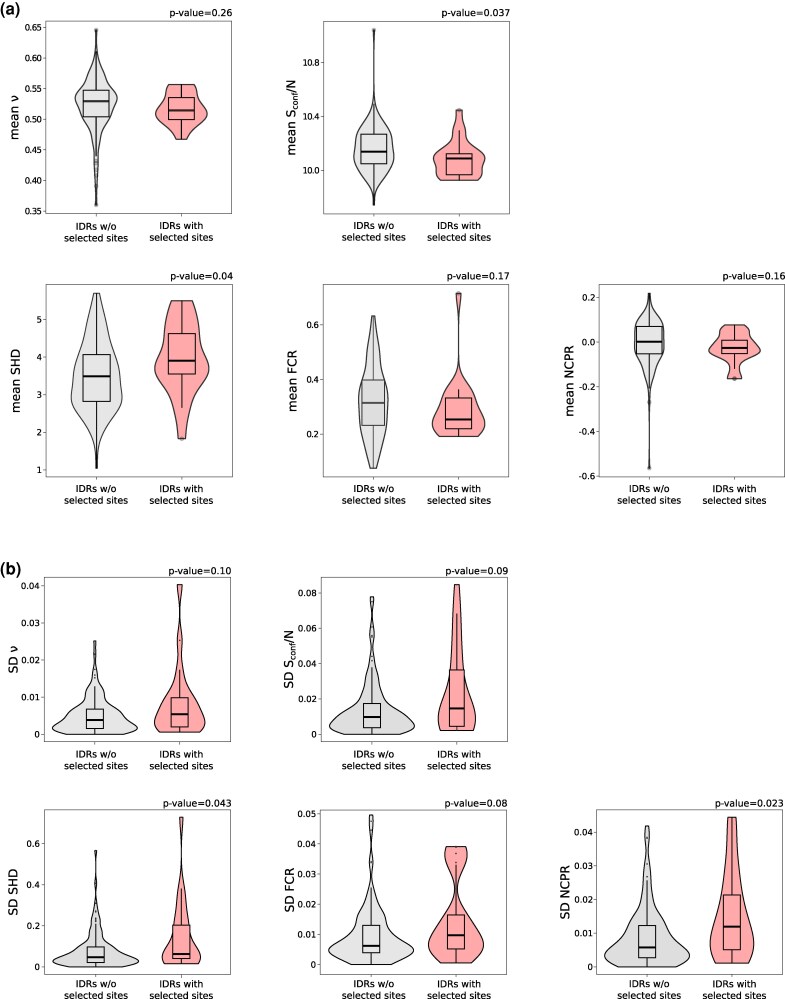
Conformational features across primate orthologous IDRs. Violin wrapping box and whiskers plots of conformational features calculated for all IDRs in TE control proteins. Plots show the mean values a) or the standard deviation b) among orthologs and colors represent IDRs that are (red) or are not (gray) targeted by positive selection. Wilcoxon rank sum test *P*-values are also reported.

We next hypothesized that the conformational properties of the IDRs (e.g. the range of conformations that are accessible to an IDR or its chain compactness) may impact selective constraint or be influenced by the positively selected residues. Although the structural properties of IDRs cannot be predicted, some ensemble properties are quantifiable and provide information on three-dimensional features and IDR function (Tesei et al. 2024). These include two length-independent parameters: the conformational entropy per residue (*S*conf/*N*) and the Flory scaling exponent (*ν*), a measure of chain compactness. These two features are nonindependent, as compact IDRs tend to have low Sconf/N (Tesei et al. 2024). We thus used a predictor based on support vector regression (SVR) models to calculate *S*conf/*N* and *ν* for all orthologous IDRs in the primate TE control proteins (Tesei et al. 2024) (Table S3). To assess whether the conformational properties influence selective constraints, for all IDRs, we also calculated the fraction of sites targeted by purifying selection. No correlation was observed between the fraction of negatively selected sites and either *S*conf/*N* or *ν* (Kendall's correlation coefficients: 0.081 and 0.027, both *P*-values > 0.05). This indicates that the selective constraint acting on IDRs is not related to their ensemble conformational features. We next compared ensemble features between IDRs that were or were not targeted by positive selection (i.e. that display at least one positively selected site or none). Interestingly, we found that positively selected IDRs have significantly lower Sconf/N and tend to be more compact, although the difference in *ν* was not statistically significant (Fig. 3a).

Previous studies showed that the amino acid composition and patterning of residues are important determinants of sequence-ensemble relationships in IDRs (Das et al. 2015; Holehouse et al. 2017; Sherry et al. 2017; Zarin et al. 2017; Beveridge et al. 2019; Zarin et al. 2019). We thus used the SVR predictor to calculate the following parameters: sequence hydropathy decoration (SHD, a measure of the patterning of hydrophobic residues) (Zheng et al. 2020), fraction of charged residues (FCR), and net charge per residue (NCPR) (Fig. 3a, Table S3). We found that positively selected IDRs have significantly higher SHD compared to the nonselected ones. They also tend to have fewer charged residues and more negative charge, although the differences are not significant (Fig. 3a). Overall, this suggests that compaction and low entropy in selected IDRs are due to the patterning of hydrophobic/aromatic residues.

To investigate how positive selection may impact IDR compositional patterns and ensemble properties, we calculated the standard deviations among primate orthologs of conformational parameters (*S*conf/*N* and *ν*) and sequence features (SHD, FCR, and NCPR). Using this approach, we obtained a single measure of feature conservation for each IDR. A comparison between positively selected and nonpositively selected IDRs indicated that the former have more variability in both ensemble features and sequence parameters than the latter, although statistical significance was only reached for SHD and NCPR (Fig. 3b). These results suggest that positive selection modulates IDR properties by introducing changes in sequence patterns and ensemble features properties, which are in turn related to function (Tesei et al. 2024).

### TE Control Genes as Targets of Positive Selection During the Recent Evolution of Human Populations

**Fig. 4.**
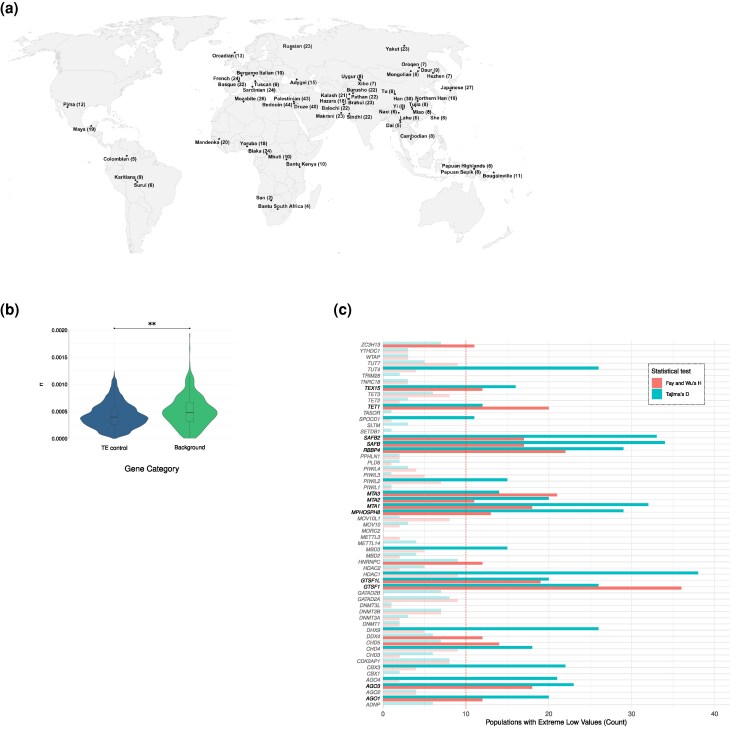
Human population genetics of TE control genes. a) Global map showing the sampling locations of the Human Genome Diversity Project populations, with the number of individuals per population indicated in parentheses. b) Nucleotide diversity (π) comparison of values for TE control genes and background genes using 1,000 subsampling. c) Distribution of TE control genes with extremely low summary statistics values across populations. The *y* axis shows genes of interest, while the *x* axis indicates the number of populations where each gene exhibits extremely low values (below the 5% threshold) for Tajima's D or Fay and Wu's H. Bars are color-coded by statistic, with transparency reduced for counts below the threshold of 10 populations (indicated by the dashed red line marks). Gene names in bold reflect those with extreme values for both D and H statistics, in at least 10 populations.

We next hypothesized that selection acting on TE control genes may have been widespread in recent human evolutionary history. Accordingly, we examined deviations from neutrality across 54 globally diverse populations represented in the Human Genome Diversity Project (HGDP) (Bergström et al. 2020) (Table S4, Fig. 4a). In order to assess whether TE-control genes bear signatures of selection, we compared nucleotide diversity (π) across windows overlapping candidate genes against a randomly sampled subset of background genes. π reflects the average number of nucleotide differences per site between two sequences and is sensitive to both historical and recent selective pressures. Our analysis revealed a significantly lower median π in candidate genes compared to background genes (Wilcoxon rank-sum test, *P*-value = 0.00538) (Fig. 4b). This indicates that, across the genome, TE-control genes tend to exhibit reduced genetic diversity, a pattern consistent with the action of purifying or recurrent positive selection.

We next examined the distribution of summary statistics (Tajima's D and Fay & Wu's H) (Tajima 1989; Fay and Wu 2000) for candidate genes across the 54 populations. Tajima's D and Fay and Wu's H assess evidence of long-term, nonrecent positive selection. Specifically, extreme values of Tajima's D identify deviations from genetic neutrality, while low values of Fay and Wu's H capture signals of high-frequency derived alleles (Nielsen et al. 2007; Ramos-Onsins et al. 2023). To account for the effect of demographic effects (Voight et al. 2006), for each test, we examined the distribution of genes with extremely ranked values (low 5%) of both statistical tests across the populations. We identified 22 and 17 genes with extremely low values of D and H, respectively, in at least 10 human populations (Fig. 4c, Table S5). Notably, 13 genes exhibited concurrent extreme values for both D and H tests in 10 or more populations, compatible with signals of positive selection of these genes across a diverse group of populations (Fig. 4c).

## Discussion

TE mobilization poses a significant fitness challenge to host genomes. As a consequence, a variety of systems have emerged to curb and silence TE activity. Just like TEs, such systems are widespread across the tree of life, and their evolution is expected to be shaped by recurrent arms races, as new TE families emerge and start amplifying. In addition to TEs, viruses can also exert a selective pressure on some of these genes, as the pathways that control retrotransposition are often also involved in the restriction of exogenous viruses (Bobadilla Ugarte et al. 2023). For instance, the HUSH complex is a crucial repressor of HIV, and it is counteracted by the Vpr and Vpx viral proteins (Chougui et al. 2018; Yurkovetskiy et al. 2018).

Here, we explored the evolution of proteins that control TEs over different timescales. Our data indicate that, in primates, a considerable fraction of TE control genes evolved under positive selection, an observation consistent with a genetic conflict scenario (Sironi et al. 2015). In particular, proteins that participate in the piRNA pathway were found to have experienced very strong selection, resulting in the identification of many sites showing fast amino acid replacement. These proteins play a central role in the silencing of retrotransposon activity in the germline, where epigenetic reprogramming results in the derepression of TEs (Almeida et al. 2022). The germline also represents the stage where the genomic conflict plays out, as new TE insertions can be transmitted through vertical inheritance only if they occur in the germline or before its specification (i.e. in the early stages of embryonic development). Indeed, the expression of many TEs seems to be restricted to various stages of gametogenesis or early embryogenesis, whereas activity in somatic tissues holds no apparent benefit to TEs (Chuong et al. 2017). That primates (like most cellular organisms) have evolved additional layers of TE control in the germline, and that these layers experience the strongest selective pressure, are thus in line with expectations under an arms race scenario. Among PIWI proteins, we found that PIWIL2, PIWIL3, and PIWIL4, but not PIWIL1, represent selection targets. Despite sequence and structural similarities, the four PIWI proteins display distinctive functional features and expression patterns. Data from rodent models indicated that *PIWIL1*, *PIWIL2*, and *PIWIL4* are expressed during male germ cell development and all three genes are essential for spermatogenesis (Iwasaki et al. 2015). PIWIL2 and PIWIL4 mainly function in transposon silencing in embryonic germ cells by associating with fetal piRNAs (Aravin et al. 2008; De Fazio et al. 2011). PIWIL1 instead has a major function in post-natal germ cells, where it associates with pachytene piRNAs and uses its slicing activity to silence TEs (Reuter et al. 2011; Wei et al. 2023). *PIWIL1* and *PIWIL3* are also expressed in oocytes and, in a hamster model, deletion of both genes determines reduced fertility, although only PIWIL1 contributes to TE repression (Hasuwa et al. 2021; Zhang et al. 2021). It is thus somewhat surprising that PIWIL1 did not show evidence of positive selection. PIWIL1 is known to load pachytene piRNAs, which are highly abundant in the adult mouse testis and largely devoid of TE sequences (Ozata et al. 2019). The precise function(s) of pachytene piRNAs are still a matter of debate. Previous works suggested that they regulate protein-coding mRNAs by promoting their deadenylation and decay (Gou et al. 2014) and that, through interaction with other proteins, the piRNA/PIWIL1 activates the translation of spermiogenic mRNAs (Dai et al. 2019). However, these pieces of evidence were not supported by more recent findings (Wu et al. 2020; Choi et al. 2021; Cecchini et al. 2026). Instead, Cecchini and coworkers showed that a small minority of pachytene piRNAs direct the endonucleolytic cleavage of few partially complementary targets, a function essential for spermatogenesis (Cecchini et al. 2026). Whereas further analyses will be required to elucidate the role of pachytene piRNAs and PIWIL1 in male fertility, it is possible that functional constraints or the necessity to interact with protein partners limit the ability of PIWIL1 to evolve in response to TE-derived selective pressures.

We should also mention that positive selection is not always determined by genetic conflicts (with either endogenous or exogenous elements), but may derive from other forces. For instance, selfish spermatogonial selection (which favors variants that increase the rate of cell division or decrease apoptosis in a given germline cell) was suggested to account for the positive selection signals in some testis-expressed genes (Nielsen et al. 2005).

Recent TE activity has also occurred in humans and a few elements, all of them retrotransposons, are still mobile in our genomes, as exemplified by polymorphic insertions (Beck et al. 2010; Osmanski et al. 2023). For instance, SVAs show evidence of relatively high recent activity (Osmanski et al. 2023), and about one hundred transposition-competent LINE1s are present in our genomes (Beck et al. 2010). We thus screened the TE control genes for evidence of positive selection in human populations, which may result from the ongoing pressure exerted by mobile elements. We detected several signals of strong positive selection across populations. In line with findings in primates, we identified as selection targets three genes (*TEX15, GTSF1*, and *GTSF1L*) that participate in the piRNA pathway (Almeida et al. 2022). In rodents, GTSF1 and GTSF1L are expressed in the male germline, where they associate with PIWI proteins to potentiate target cleavage (Arif et al. 2022). Deletion of *Gtsf1* results in male sterility, whereas knockout of *Gtsf1l* does not impair spermatogenesis or retrotransposon repression in mice, although more subtle phenotypic changes cannot be excluded (Takemoto et al. 2016; Yoshimura et al. 2018). Whereas this may suggest functional redundancy, more recent evidence indicates that GTSF1 and GTSF1L (as well as a third paralog, GTSF2) may have specialized roles in enhancing target cleavage by specific PIWI proteins (Arif et al. 2022). If a similar specialization has occurred in primates, the signatures of selection we detected may reflect fine-tuning of protein–protein interactions or expression patterns to match those of distinct PIWI proteins. Alternatively, these genes may have acquired divergent functions or expression profiles in primates. Experimental studies will be required to distinguish between these possibilities. The third component of the piRNA pathway, TEX15, is also associated with male sterility in both mice and humans (Yang et al. 2008; He et al. 2013). In the male germline, TEX15 interacts with PIWIL1 and PIWIL2 and is required for piRNA-directed de novo DNA methylation (Schöpp et al. 2020; Yang et al. 2020). TEX15 has been a target of positive selection across different evolutionary timescales—during primate evolution and more recently in human populations. It will be particularly informative to determine, through population-level analyses, whether selection acting on TEX15 or other components of the piRNA pathway is linked to male reproductive fitness.

With the exclusion of *TEX15*, however, the genes that experienced the strongest selective pressure during the recent evolution of human populations did not overlap with those undergoing fast evolution in primates. Interestingly, four of the 13 genes with strong evidence of selection encode components of the NuRD complex. In the context of TE suppression, this complex interacts with KAP1 and mediates de novo heterochromatin formation, thus transcriptionally suppressing LTR retrotransposons (Rowe et al. 2010). However, NuRD plays additional functions in the cell and its activity is essential for the regulation of gene expression, cell fate specification, and cell cycle progression (Hoffmann and Spengler 2019). As a consequence, deregulation of NuRD has been associated with cancer and neurodevelopmental disorders (Lai and Wade 2011; Hoffmann and Spengler 2019). Moreover, NuRD is the target of several infectious viruses, most notably herpesviruses (Savaryn et al. 2013; Salamun et al. 2019; Naik et al. 2020), that hijack transcriptional regulation. It is thus possible that the selective pressures acting in human populations on NuRD components are not directly linked to its TE-silencing activity. It is also worth noting that variants in some of the genes (*MTA1, RBBP4, AGO1, SAFB2,* and *TET1*) showing strong evidence of positive selection in human populations are associated to anthropometric traits (waist-to-hip ratio adjusted for BMI, height) (https://www.ebi.ac.uk/gwas/home). In turn, such traits were shown to carry evidence of polygenic adaptation in humans (Turchin et al. 2012; Robinson et al. 2015; Polimanti et al. 2016; Guo et al. 2018), raising the possibility that the signals we detected are related to the pleiotropic functions of these genes.

Given that their biological functions often involve nucleic acid binding, the proteins encoded by the genes we analyzed are particularly rich in IDRs (Liu et al. 2006; Zhao et al. 2021), which pose challenges in their analysis: they are, in general, poorly conserved and, because they lack stable secondary or tertiary structures, the biological and biochemical functions of IDRs are difficult to predict (Holehouse and Kragelund 2024). As a consequence, IDRs and their evolutionary trajectories remain under-investigated (Tesei et al. 2024). We found that signals of positive selection are significantly enriched within the disordered portions of proteins involved in TE control. Whereas this finding is in line with a previous study of human IDRs, it opens the question of how changes in IDRs can promote adaptation and which biological functions are affected by such changes (Afanasyeva et al. 2018). We thus applied recently developed methods to investigate IDR sequence-ensemble properties in relation to evolutionary parameters. As growing evidence indicates that chain compaction and conformational entropy are relevant descriptors of IDR function, we first asked whether these parameters correlate with the level of selective constraint, measured as the fraction of sites experiencing purifying selection. We found no evidence of correlation, which was somewhat unexpected because previous data showed that human pathogenic variants are more likely to be located in IDRs with low conformational entropy compared to benign variants (Tesei et al. 2024), suggesting that such IDRs are less tolerant to change. It is however possible that human IDRs are heterogeneous with respect to the relationship between tolerance and entropy, which may depend on protein function. Analysis of positive selection signals, however, showed that they are preferentially located in regions of low conformational entropy. We thus hypothesized that, by altering the sequence composition or patterning of the IDRs, the positively selected sites might modulate the conformational ensemble features and, hence, IDR functions. By calculating across-orthologs variance, we verified this hypothesis, although statistical significance was often borderline, most likely due to the small number of comparisons. Experimental investigation or molecular dynamic simulations will be required to determine the effect of positive selection on IDR function in these proteins. Nonetheless, our data show that the evolutionary trajectories of IDRs may be linked to their conformational characteristics, exactly in the same way as structured domains often evolve by changes that affect the solvent-exposed surface rather than the inner hydrophobic cores. This conclusion is reminiscent of findings on IDRs in proteins that derived from retrotransposon domestication, suggesting that it may represent a general feature of IDR evolution in mammals (Cagliani et al. 2024b). Unfortunately, the specific functions of IDRs in these TE control proteins are mostly unknown and, because they cannot be investigated in classical structural analyses (IDRs typically fail to be solved in crystals), their relative contribution to the formation of protein–RNA complexes is unexplored. In general, IDRs are known to often mediate multivalent inter-molecular interactions (Holehouse and Kragelund 2024). Thus, an interesting possibility is that selected changes in IDRs modulate the interactions among protein–protein and protein–RNA complexes, eventually contributing to the regulation of silencing activities. IDRs are also known to represent common targets of post-translational modifications (PTMs) and to often host eukaryotic linear motifs (ELMs, short linear sequences important for protein regulation) (Holehouse and Kragelund 2024). Both PTMs and ELMs confer an additional layer of protein regulation and may be affected by the selected sites. Experimental analyses using site-directed mutagenesis of the positively selected sites will be required to shed light on their role in protein regulation, complex formation, and TE silencing.

In summary, we performed an evolutionary analysis of TE control systems across different timescales. Our working hypothesis was that the evolution of such systems has been dynamic, as a result of genetic conflicts with TEs. In line with this scenario, we show that positive selection drove the evolution of TE control proteins in primates and human populations. This dual-timescale approach reveals that different genes and pathways, such as the piRNA pathway in primates and the NuRD complex in humans, have been under distinct selective pressures over time. This provides a more complete and dynamic picture of the genomic conflict with transposable elements. Thus, our data add up to similar observations in other organisms, including *Drosophila* and fish, indicating that TE control proteins are fast evolving (Obbard et al. 2009; Lee and Langley 2012; Simkin et al. 2013; Yi et al. 2014; Parhad and Theurkauf 2019). As noted elsewhere, though, a major open question is to determine the mechanistic details driving the conflict (Blumenstiel et al. 2016). TEs are not known to encode antagonists of proteins involved in their repression and the capacity of TEs to evolve evasion mechanisms of the silencing machinery may be limited (Blumenstiel et al. 2016). TE diversity has been regarded as a driver of adaptation of the piRNA pathway (Lee and Langley 2012; Yi et al. 2014). However, an analysis in *Drosophila* showed that the rates of evolution in species with high TE burden and diversity are slower than in species with more abundant and diverse TEs (Castillo et al. 2011). It was thus suggested that evolution of the piRNA pathway (and possibly other proteins involved in TE control) results from a trade-off between genome defense and the costs of off-target gene silencing—that is a balance between sensitivity and specificity (Blumenstiel et al. 2016). To test this hypothesis, it would be useful to determine whether amino acid changes at the positively selected sites modulate sensitivity or off-target effects. In this respect, it is also worth mentioning that, whereas in the analysis of primate genes we focused on protein sequence variation, the selection signatures that we detected in human populations are likely to be primarily driven by regulatory changes (Grossman et al. 2013). Again based on the *Drosophila* model, it was previously suggested that adaptation to high TE burden can also be obtained by increased expression of the piRNA machinery (Castillo et al. 2011). These observations might also explain why we detected limited overlap in the gene sets that were selected in primates and during the evolution of human populations. Possibly, different adaptive mechanisms drive the evolution of distinct genes or the response to specific TE lineages. It will thus be extremely important to determine whether the selected variants modulate the level or timing of expression of TE control genes in humans. Also, the fine mapping of the selection signals in humans will provide a list of candidate variants for functional analyses.

## Materials and Methods

### Gene Selection

The list of 60 coding genes involved in TE control was derived from previous reviews and individual studies (Aktaş et al. 2017; Almeida et al. 2022; Wang et al. 2023; Zhao et al. 2023; Ilık et al. 2024) (Table S1). KZFPs were not included because (i) they were previously analyzed and (ii) their fast evolution in terms of gene copies makes it difficult to reconstruct orthology relationships (Thomas and Schneider 2011; Jacobs et al. 2014; Kosuge et al. 2024).

### Primate Sequence Retrieval and Positive Selection Analysis

We obtained primate orthologous coding sequences from the NCBI database (http://www.ncbi.nlm.gov). Specifically, we exploited data from the NCBI's Eukaryotic Genome Annotation Pipeline to derive primate one-to-one orthologs (NCBI Orthologs) (Oh et al. 2025). Primate genes carrying premature stop codons or with a low sequence coverage were excluded. A list of primate species analyzed for each gene is reported in Table S1. To evaluate the quality and the completeness of the analyzed genomes, different assembly statistics were retrieved from NCBI, confirming an overall high quality (Table S1).

The RevTrans 2.0 utility was used to generate multiple sequence alignments (MSAs) using MAFFT v6.240 as an aligner (Wernersson 2003; Katoh and Standley 2013). The resulting alignments were manually trimmed and adjusted to exclude segments with gaps in most species and to include only well-aligning regions. MSAs were analyzed for the presence of recombination signals using Genetic Algorithm Recombination Detection (GARD, parameters: general discrete model, three rate classes) (Pond et al. 2006). GARD is a genetic algorithm based on phylogenetic incongruence among alignment segments, which detects the best-fit number and location of recombination breakpoints. When evidence of recombination was detected, the coding alignment was split based on the recombination breakpoints and sub-regions (of at least 50 nucleotides) were used as input for subsequent molecular evolution analyses. Recombination breakpoints were identified for *MOV10L1*, *PIWIL3*, and *TNRC18* alignments (Table S2).

Phylogenetic trees were reconstructed using the phyML program (version 3.1) with a General Time Reversible (GTR) model plus gamma-distributed rates and four substitution rate categories with a fixed proportion of invariable sites (Guindon et al. 2009). The MSAs and phyML trees were used as inputs to perform evolutionary analysis. To detect positive selection, the codon-based *codeml* program implemented in the PAML (Phylogenetic Analysis by Maximum Likelihood) suite was applied (Yang 2007), using the F3x4 codon frequencies model (codon frequencies estimated from the nucleotide frequencies in the data at each codon site) (Yang 1997; Yang 2007). A model (M8, positive selection model) that allows a class of sites to evolve with dN/dS > 1 was compared to two models (M7 and M8a, neutral models) that do not allow dN/dS > 1. To assess statistical significance, twice the difference of the likelihood (ΔlnL) for each model (M8a vs. M8 and M7 vs. M8) is compared to a *χ*^2^ distribution (1 and 2 degrees of freedom for M8a vs. M8 and M7 vs. M8 comparisons, respectively). To be conservative and to obtain robust results, we called a gene as a target of positive selection only if it was detected by both M7 versus M8 and M8a versus M8 comparisons.

In order to identify specific sites subject to positive selection, we applied four different methods: (i) the Bayes Empirical Bayes (BEB) analysis (with a cutoff of 0.90), which calculates the posterior probability that each codon is from the positive selection site class (under M8 model) (Anisimova et al. 2002); (ii) Fast Unbiased Bayesian AppRoximation (FUBAR) (with a cutoff of 0.90), an approximate hierarchical Bayesian method that generates an unconstrained distribution of selection parameters to estimate the posterior probability of positive diversifying selection at each site in a given alignment (Murrell et al. 2013); (iii) Mixed Effects Model of Evolution (MEME) (with a *P*-value cutoff < 0.1), which allows the distribution of dN/dS to vary from site to site and from branch to branch at a site (Murrell et al. 2012); (iv) Fixed Effects Likelihood (FEL) (with a *P*-value cutoff < 0.1), a maximum-likelihood (ML) approach to infer dN/dS on a per-site basis, assuming that the selection pressure for each site is constant along the entire phylogeny (Kosakovsky Pond and Frost 2005). To be conservative and to limit false positives, only sites detected using at least two methods were considered as positive selection targets.

The single-likelihood ancestor counting (SLAC) method was applied to identify sites under negative/purifying selection (Kosakovsky Pond and Frost 2005). GARD, FUBAR, MEME, FEL, and SLAC analyses were run locally through the HyPhy suite V2.5.29 (Pond et al. 2005).

### Analysis and Prediction of Protein Three-Dimensional Structures

The structure of PIWIL2 in complex with piRNA was derived from PDB (https://www.wwpdb.org/, ID:7yfx), whereas the structural models of human PIWIL3 and PIWIL4 were obtained from the AlphaFold database. TM-align (https://zhanggroup.org/TM-align/) (Zhang et al. 2005) was run for pairwise structure alignments.

AlphaFold3 (Abramsom et al. 2024), through the AlphaFold Server (https://alphafoldserver.com/), was used to model the structure of the human PIWIL4-GTSF1-MAEL-piRNA-target RNA-TDRD9 complex. 0.8×ipTM + 0.2×pTM > 0.7 was applied as a confidence metric for quality estimation (Shor et al. 2024).

3D structures were rendered using PyMOL (The PyMOL Molecular Graphics System, Version 1.8.4.0; Schrödinger, LLC).

### Prediction of Intrinsically Disordered Regions

The sequences of reviewed (Swiss-Prot) canonical proteins (20,420) of the reference human proteome (UP000005640) were retrieved from Uniprot (https://www.uniprot.org/). Intrinsically disordered regions (IDRs) were estimated using the Metapredict V2 tool (Emenecker et al. 2021, 2022), that defines which residues from a protein sequence are disordered by applying a deep-learning algorithm based on a consensus score calculated from eight different predictors (Emenecker et al. 2021). Metapredict V2 was run using default parameters and consecutive disordered stretches equal to or longer than 30 residues were labeled as IDRs. For primate genes, IDRs were kept only if the same orthologous region was predicted in at least 50% of the species analyzed. Disorder annotations were also derived from MobiDB (Piovesan et al. 2025). This database aggregates disorder information from literature, experimental data, and predictions for all known proteins recorded in Uniprot. The consensus disorder annotations were used in our analysis.

### Analysis of IDR Conformational Properties and Sequence Patterns

The conformational entropy per residue (Sconf/N) and the Flory scaling exponent (*ν*) were calculated for all analyzed IDRs. *S*conf/*N* is a measure of the landscape of different structures accessible to an IDR. *ν* derives from the scaling laws of polymers that describe how chain dimensions vary as a function of chain length (Flory and Volkenstein 1969). Both parameters were estimated using a Colab notebook (https://colab.research.google.com/github/KULL-Centre/_2023_Tesei_IDRome/blob/main/IDR_SVR_predictor.ipynb) (Tesei and Lindorff-Larsen 2022; Tesei et al. 2024), which uses a support vector regression model trained on simulations performed using the CALVADOS model (Tesei et al. 2021; Tesei and Lindorff-Larsen 2022). The same SVR predictor was used to derive other sequence measures: SHD (sequence hydropathy decoration) (Zheng et al. 2020), FCR (the fraction of charged residue), and NCPR (net charge per residue).

### Population Genetics Analyses

We conducted our analysis on a dataset of variant call format (VCF) files comprising genetic variation data of autosomal chromosomes from 54 distinct human populations and 828 individuals from the Human Genome Diversity Panel (Bergström et al. 2020) (Table S4). VCF files were further indexed and filtered for minimum quality parameters (-minQ/-minMapQ < 20) using BCFtools and SAMtools (Li 2011; Danecek et al. 2021).

To assess potential signals of selection, we compared nucleotide diversity (*π*) between candidate TE-control genes and background genes. The latter comprised 18,161 annotated protein-coding genes in the human reference genome (GRCh38), providing a genome-wide baseline for comparison. This approach allowed us to evaluate whether TE-control genes exhibit distinct patterns of genetic diversity relative to the broader set of coding regions. Genetic diversity values were calculated per genomic window. We then performed a Wilcoxon rank-sum test on the median *π* values between candidate genes and a randomly sampled subset of equal size from the background genes, repeated over 1,000 iterations to ensure robustness. For each population, we also calculated two summary statistics of genetic diversity: Tajima's D and Fay's H. Tajima's D, Fay and Wu's H and nucleotide diversity (*π*) were all calculated in sliding windows, with a window size of 50 kilobases (kb) with a step size of 10 kb, using the software ANGSD (Korneliussen et al. 2014). We ranked the values for each summary statistics, and each population separately, then assigned the minimum rank value to each gene, considering all the windows encompassing its genomic coordinates.

We found no significant difference in lengths between candidate genes and the rest of the human genes. To test for signals of selection, we ranked the calculated summary statistics within each population. For each gene, we identified windows that fell into the extreme 5% of ranked values for Tajima's D and Fay and Wu's H (Table S5). We then tagged the TE control genes that fell in those lower values and visualized the frequency with which those genes are found across the multiple populations. The analysis was conducted using RStudio and R version 4.2.2 and utilized the ggplot2 package (Wickham 2016) for visualization.

### Statistical Analysis

To evaluate whether positively selected sites are enriched in IDRs, we applied a binomial test, using the counts of sites falling in IDRs as the number of successes, the number of total selected sites as trials, and the ratio between all IDR lengths divided by the length of all analyzed regions as the probability of success. Wilcoxon rank sum and Kendall's correlation tests were performed in the R v.4.0.5 environment.

## Supplementary Material

evag059_Supplementary_Data

## Data Availability

The data underlying this article are available in the article and in its Online Supplementary Material. Multiple sequence alignments, trees, and IDR sequences are available in Zenodo, at https://dx.doi.org/10.5281/zenodo.17433528.

## References

[evag059-B1] Abramson J, et al Accurate structure prediction of biomolecular interactions with AlphaFold 3. Nature. 2024:630:493–500. 10.1038/s41586-024-07487-w. 38718835 PMC11168924

[evag059-B2] Afanasyeva A, Bockwoldt M, Cooney CR, Heiland I, Gossmann TI. Human long intrinsically disordered protein regions are frequent targets of positive selection. Genome Res. 2018:28:975–982. 10.1101/gr.232645.117. 29858274 PMC6028134

[evag059-B3] Aktaş T, et al DHX9 suppresses RNA processing defects originating from the Alu invasion of the human genome. Nature. 2017:544:115–119. 10.1038/nature21715. 28355180

[evag059-B4] Almeida MV, Vernaz G, Putman ALK, Miska EA. Taming transposable elements in vertebrates: from epigenetic silencing to domestication. Trends Genet. 2022:38:529–553. 10.1016/j.tig.2022.02.009. 35307201

[evag059-B5] Almojil D, et al The structural, functional and evolutionary impact of transposable elements in eukaryotes. Genes (Basel). 2021:12:918. 10.3390/genes12060918. 34203645 PMC8232201

[evag059-B6] Anisimova M, Bielawski JP, Yang Z. Accuracy and power of Bayes prediction of amino acid sites under positive selection. Mol Biol Evol. 2002:19:950–958. 10.1093/oxfordjournals.molbev.a004152. 12032251

[evag059-B7] Aravin AA, et al A piRNA pathway primed by individual transposons is linked to de novo DNA methylation in mice. Mol Cell. 2008:31:785–799. 10.1016/j.molcel.2008.09.003. 18922463 PMC2730041

[evag059-B8] Arif A, et al GTSF1 accelerates target RNA cleavage by PIWI-clade Argonaute proteins. Nature. 2022:608:618–625. 10.1038/s41586-022-05009-0. 35772669 PMC9385479

[evag059-B9] Beck CR, et al LINE-1 retrotransposition activity in human genomes. Cell. 2010:141:1159–1170. 10.1016/j.cell.2010.05.021. 20602998 PMC3013285

[evag059-B10] Bergström A, et al Insights into human genetic variation and population history from 929 diverse genomes. Science. 2020:367:eaay5012. 10.1126/science.aay5012. 32193295 PMC7115999

[evag059-B11] Beveridge R, et al Ion mobility mass spectrometry uncovers the impact of the patterning of oppositely charged residues on the conformational distributions of intrinsically disordered proteins. J Am Chem Soc. 2019:141:4908–4918. 10.1021/jacs.8b13483. 30823702 PMC6488185

[evag059-B12] Blum M, et al InterPro: the protein sequence classification resource in 2025. Nucl Acids Res. 2025:53:D444–D456. 10.1093/nar/gkae1082. 39565202 PMC11701551

[evag059-B13] Blumenstiel JP, Erwin AA, Hemmer LW. What drives positive selection in the Drosophila piRNA machinery? The genomic autoimmunity hypothesis. Yale J Biol Med. 2016:89:499–512. 28018141 PMC5168828

[evag059-B14] Bobadilla Ugarte P, Barendse P, Swarts DC. Argonaute proteins confer immunity in all domains of life. Curr Opin Microbiol. 2023:74:102313. 10.1016/j.mib.2023.102313. 37023508

[evag059-B15] Bourque G, et al Ten things you should know about transposable elements. Genome Biol. 2018:19:199. 10.1186/s13059-018-1577-z. 30454069 PMC6240941

[evag059-B16] Cagliani R, et al Evolutionary analysis of ZAP and its cofactors identifies intrinsically disordered regions as central elements in host-pathogen interactions. Comput Struct Biotechnol. 2024a:23:3143–3154. 10.1016/j.csbj.2024.07.022. PMC11372611 39234301

[evag059-B17] Cagliani R, et al Evolution of virus-like features and intrinsically disordered regions in retrotransposon-derived mammalian genes. Mol Biol Evol. 2024b:41. 10.1093/molbev/msae154. PMC11299033 39101471

[evag059-B18] Castillo DM, Mell JC, Box KS, Blumenstiel JP. Molecular evolution under increasing transposable element burden in Drosophila: a speed limit on the evolutionary arms race. BMC Evol Biol. 2011:11:258. 10.1186/1471-2148-11-258. 21917173 PMC3185285

[evag059-B19] Cecchini K, et al Cleavage of mRNAs by a minority of pachytene piRNAs improves sperm fitness. Nature. 2026. 10.1038/s41586-026-10102-9. PMC13061629 41639461

[evag059-B20] Choi H, Wang Z, Dean J. Sperm acrosome overgrowth and infertility in mice lacking chromosome 18 pachytene piRNA. PLoS Genet. 2021:17:e1009485. 10.1371/journal.pgen.1009485. 33831001 PMC8057611

[evag059-B21] Chougui G, et al HIV-2/SIV viral protein X counteracts HUSH repressor complex. Nat Microbiol. 2018:3:891–897. 10.1038/s41564-018-0179-6. 29891865

[evag059-B22] Chuong EB, Elde NC, Feschotte C. Regulatory activities of transposable elements: from conflicts to benefits. Nat Rev Genet. 2017:18:71–86. 10.1038/nrg.2016.139. 27867194 PMC5498291

[evag059-B23] Dai P, et al A translation-activating function of MIWI/piRNA during mouse spermiogenesis. Cell. 2019:179:1566–1581.e16. 10.1016/j.cell.2019.11.022. 31835033 PMC8139323

[evag059-B24] Danecek P, et al Twelve years of SAMtools and BCFtools. GigaScience. 2021:10:giab008. 10.1093/gigascience/giab008. 33590861 PMC7931819

[evag059-B25] Das RK, Ruff KM, Pappu RV. Relating sequence encoded information to form and function of intrinsically disordered proteins. Curr Opin Struct Biol. 2015:32:102–112. 10.1016/j.sbi.2015.03.008. 25863585 PMC4512920

[evag059-B26] De D, et al A conserved PIWI silencing complex detects piRNA-target engagement. Mol Cell. 2025:85:3275–3287.e7. 10.1016/j.molcel.2025.08.010. 40912244 PMC12416740

[evag059-B27] De Fazio S, et al The endonuclease activity of Mili fuels piRNA amplification that silences LINE1 elements. Nature. 2011:480:259–263. 10.1038/nature10547. 22020280

[evag059-B28] De Koning APJ, Gu W, Castoe TA, Batzer MA, Pollock DD. Repetitive elements may comprise over two-thirds of the human genome. PLoS Genet. 2011:7:e1002384. 10.1371/journal.pgen.1002384. 22144907 PMC3228813

[evag059-B29] Doolittle WF, Sapienza C. Selfish genes, the phenotype paradigm and genome evolution. Nature. 1980:284:601–603. 10.1038/284601a0. 6245369

[evag059-B30] Emenecker RJ, Griffith D, Holehouse AS. Metapredict: a fast, accurate, and easy-to-use predictor of consensus disorder and structure. Biophys J. 2021:120:4312–4319. 10.1016/j.bpj.2021.08.039. 34480923 PMC8553642

[evag059-B31] Emenecker RJ, Griffith D, Holehouse AS. 2022 June 09. Metapredict V2: an update to metapredict, a fast, accurate, and easy-to-use predictor of consensus disorder and structure [preprint]. bioRxiv 494887. 10.1101/2022.06.06.494887 [accessed 2024 May 16]. PMC8553642 34480923

[evag059-B32] Fay JC, Wu C-I. Hitchhiking under positive Darwinian selection. Genetics. 2000:155:1405–1413. 10.1093/genetics/155.3.1405. 10880498 PMC1461156

[evag059-B33] Feusier J, et al Pedigree-based estimation of human mobile element retrotransposition rates. Genome Res. 2019:29:1567–1577. 10.1101/gr.247965.118. 31575651 PMC6771411

[evag059-B34] Finnegan DJ . Eukaryotic transposable elements and genome evolution. Trends Genet. 1989:5:103–107. 10.1016/0168-9525(89)90039-5. 2543105

[evag059-B35] Flory PJ, Volkenstein M. Statistical mechanics of chain molecules. Biopolymers. 1969:8:699–700. 10.1002/bip.1969.360080514.

[evag059-B36] Gou L-T, et al Pachytene piRNAs instruct massive mRNA elimination during late spermiogenesis. Cell Res. 2014:24:680–700. 10.1038/cr.2014.41. 24787618 PMC4042167

[evag059-B37] Grossman SR, et al Identifying recent adaptations in large-scale genomic data. Cell. 2013:152:703–713. 10.1016/j.cell.2013.01.035. 23415221 PMC3674781

[evag059-B38] Guindon S, Delsuc F, Dufayard JF, Gascuel O. Estimating maximum likelihood phylogenies with PhyML. Methods Mol Biol (Clifton, NJ). 2009:537:113–137. 10.1007/978-1-59745-251-9_6. 19378142

[evag059-B39] Guo J, et al Global genetic differentiation of complex traits shaped by natural selection in humans. Nat Commun. 2018:9:1865. 10.1038/s41467-018-04191-y. 29760457 PMC5951811

[evag059-B40] Hancks DC, Kazazian HH. Roles for retrotransposon insertions in human disease. Mob DNA. 2016:7:9. 10.1186/s13100-016-0065-9. 27158268 PMC4859970

[evag059-B41] Hasuwa H, et al Production of functional oocytes requires maternally expressed PIWI genes and piRNAs in golden hamsters. Nat Cell Biol. 2021:23:1002–1012. 10.1038/s41556-021-00745-3. 34489571

[evag059-B42] Haudiquet M, De Sousa JM, Touchon M, Rocha EPC. Selfish, promiscuous and sometimes useful: how mobile genetic elements drive horizontal gene transfer in microbial populations. Philos Trans R Soc B. 2022:377:20210234. 10.1098/rstb.2021.0234. PMC9393566 35989606

[evag059-B43] He X-J, et al TEX15 causes spermatogenesis disorder by effecting meiotic recombination. Fertil Steril. 2013:100:S219. 10.1016/j.fertnstert.2013.07.1311.

[evag059-B44] Hoffmann A, Spengler D. Chromatin remodeling complex NuRD in neurodevelopment and neurodevelopmental disorders. Front Genet. 2019:10:682. 10.3389/fgene.2019.00682. 31396263 PMC6667665

[evag059-B45] Holehouse AS, Das RK, Ahad JN, Richardson MOG, Pappu RV. CIDER: resources to analyze sequence-ensemble relationships of intrinsically disordered proteins. Biophys J. 2017:112:16–21. 10.1016/j.bpj.2016.11.3200. 28076807 PMC5232785

[evag059-B46] Holehouse AS, Kragelund BB. The molecular basis for cellular function of intrinsically disordered protein regions. Nat Rev Mol Cell Biol. 2024:25:187–211. 10.1038/s41580-023-00673-0. 37957331 PMC11459374

[evag059-B47] Hurst LD, Atlan A, Bengtsson BO. Genetic conflicts. Q Rev Biol. 1996:71:317–364. 10.1086/419442. 8828237

[evag059-B48] Ilık İA, et al Autonomous transposons tune their sequences to ensure somatic suppression. Nature. 2024:626:1116–1124. 10.1038/s41586-024-07081-0. 38355802 PMC10901741

[evag059-B49] Iwasaki YW, Siomi MC, Siomi H. PIWI-interacting RNA: its biogenesis and functions. Annu Rev Biochem. 2015:84:405–433. 10.1146/annurev-biochem-060614-034258. 25747396

[evag059-B50] Jacobs FMJ, et al An evolutionary arms race between KRAB zinc-finger genes ZNF91/93 and SVA/L1 retrotransposons. Nature. 2014:516:242–245. 10.1038/nature13760. 25274305 PMC4268317

[evag059-B51] Jangam D, Feschotte C, Betrán E. Transposable element domestication as an adaptation to evolutionary conflicts. Trends Genet. 2017:33:817–831. 10.1016/j.tig.2017.07.011. 28844698 PMC5659911

[evag059-B52] Joly-Lopez Z, Bureau TE. Exaptation of transposable element coding sequences. Curr Opin Genet Dev. 2018:49:34–42. 10.1016/j.gde.2018.02.011. 29525543

[evag059-B53] Kapitonov VV, Jurka J. Self-synthesizing DNA transposons in eukaryotes. Proc Natl Acad Sci U S A. 2006:103:4540–4545. 10.1073/pnas.0600833103. 16537396 PMC1450207

[evag059-B54] Katoh K, Standley DM. MAFFT multiple sequence alignment software version 7: improvements in performance and usability. Mol Biol Evol. 2013:30:772–780. 10.1093/molbev/mst010. 23329690 PMC3603318

[evag059-B55] Korneliussen TS, Albrechtsen A, Nielsen R. ANGSD: analysis of next generation sequencing data. BMC Bioinformatics. 2014:15:356. 10.1186/s12859-014-0356-4. 25420514 PMC4248462

[evag059-B56] Kosakovsky Pond SL, Frost SDW. Not so different after all: a comparison of methods for detecting amino acid sites under selection. Mol Biol Evol. 2005:22:1208–1222. 10.1093/molbev/msi105. 15703242

[evag059-B57] Kosuge M, Ito J, Hamada M. Landscape of evolutionary arms races between transposable elements and KRAB-ZFP family. Sci Rep. 2024:14:23358. 10.1038/s41598-024-73752-7. 39375372 PMC11458898

[evag059-B58] Lai AY, Wade PA. Cancer biology and NuRD: a multifaceted chromatin remodelling complex. Nat Rev Cancer. 2011:11:588–596. 10.1038/nrc3091. 21734722 PMC4157524

[evag059-B59] Lasserre A et al 2023 March 29. MORC2 restriction factor silences HIV proviral expression [preprint]. bioRxiv 534756 [accessed 2024 Dec 11]. 10.1101/2023.03.29.534756

[evag059-B60] Lee YCG, Langley CH. Long-term and short-term evolutionary impacts of transposable elements on *Drosophila*. Genetics. 2012:192:1411–1432. 10.1534/genetics.112.145714. 22997235 PMC3512147

[evag059-B61] Li H . A statistical framework for SNP calling, mutation discovery, association mapping and population genetical parameter estimation from sequencing data. Bioinformatics (Oxford, England). 2011:27:2987–2993. 10.1093/bioinformatics/btr509. 21903627 PMC3198575

[evag059-B62] Li Z, et al Mammalian PIWI–piRNA–target complexes reveal features for broad and efficient target silencing. Nat Struct Mol Biol. 2024:31:1222–1231. 10.1038/s41594-024-01287-6. 38658622

[evag059-B63] Liu J, et al Intrinsic disorder in transcription factors. Biochemistry. 2006:45:6873–6888. 10.1021/bi0602718. 16734424 PMC2538555

[evag059-B64] Mendez-Dorantes C, Burns KH. LINE-1 retrotransposition and its deregulation in cancers: implications for therapeutic opportunities. Genes Dev. 2023:37:948–967. 10.1101/gad.351051.123. 38092519 PMC10760644

[evag059-B65] Molaro A, Malik HS, Bourc’his D. Dynamic evolution of de novo DNA methyltransferases in rodent and primate genomes. Mol Biol Evol. 2020:37:1882–1892. 10.1093/molbev/msaa044. 32077945 PMC7306680

[evag059-B66] Murrell B, et al Detecting individual sites subject to episodic diversifying selection. PLoS Genet. 2012:8:e1002764. 10.1371/journal.pgen.1002764. 22807683 PMC3395634

[evag059-B67] Murrell B, et al FUBAR: a fast, unconstrained Bayesian approximation for inferring selection. Mol Biol Evol. 2013:30:1196–1205. 10.1093/molbev/mst030. 23420840 PMC3670733

[evag059-B68] Naik NG, et al Epigenetic factor siRNA screen during primary KSHV infection identifies novel host restriction factors for the lytic cycle of KSHV. PLoS Pathog. 2020:16:e1008268. 10.1371/journal.ppat.1008268. 31923286 PMC6977772

[evag059-B69] Nielsen R, et al A scan for positively selected genes in the genomes of humans and chimpanzees. PLoS Biol. 2005:3:e170. 10.1371/journal.pbio.0030170. 15869325 PMC1088278

[evag059-B70] Nielsen R, Hellmann I, Hubisz M, Bustamante C, Clark AG. Recent and ongoing selection in the human genome. Nat Rev Genet. 2007:8:857–868. 10.1038/nrg2187. 17943193 PMC2933187

[evag059-B71] Obbard DJ, Gordon KHJ, Buck AH, Jiggins FM. The evolution of RNAi as a defence against viruses and transposable elements. Philos Trans R Soc B. 2009:364:99–115. 10.1098/rstb.2008.0168. PMC2592633 18926973

[evag059-B72] Oh D-H, et al NCBI orthologs: public resource and scalable method for computing high-precision orthologs across eukaryotic genomes. J Mol Evol. 2025:93:843–859. 10.1007/s00239-025-10268-2. 40996513 PMC12756343

[evag059-B73] Osmanski AB, et al Insights into mammalian TE diversity through the curation of 248 genome assemblies. Science. 2023:380:eabn1430. 10.1126/science.abn1430. 37104570 PMC11103246

[evag059-B74] Ozata DM, Gainetdinov I, Zoch A, O’Carroll D, Zamore PD. PIWI-interacting RNAs: small RNAs with big functions. Nat Rev Genet. 2019:20:89–108. 10.1038/s41576-018-0073-3. 30446728

[evag059-B75] Parhad SS, Theurkauf WE. Rapid evolution and conserved function of the piRNA pathway. Open Biol. 2019:9:180181. 10.1098/rsob.180181. 30958115 PMC6367137

[evag059-B76] Piovesan D, et al MOBIDB in 2025: integrating ensemble properties and function annotations for intrinsically disordered proteins. Nucleic Acids Res. 2025:53:D495–D503. 10.1093/nar/gkae969. 39470701 PMC11701742

[evag059-B77] Polimanti R, Yang BZ, Zhao H, Gelernter J. Evidence of polygenic adaptation in the systems genetics of anthropometric. PLoS One. 2016:11:e0160654. 10.1371/journal.pone.0160654. 27537407 PMC4990182

[evag059-B78] Pond SLK, Frost SDW, Muse SV. Hyphy: hypothesis testing using phylogenies. Bioinformatics. 2005:21:676–679. 10.1093/bioinformatics/bti079. 15509596

[evag059-B79] Pond SLK, Posada D, Gravenor MB, Woelk CH, Frost SDW. Automated phylogenetic detection of recombination using a genetic algorithm. Mol Biol Evol. 2006:23:1891–1901. 10.1093/molbev/msl051. 16818476

[evag059-B80] Portell-Montserrat J, et al Target RNA recognition drives PIWI∗ complex assembly for transposon silencing. Mol Cell. 2025:85:3288–3305.e6. 10.1016/j.molcel.2025.08.007. 40912245

[evag059-B81] Ramos-Onsins SE, Marmorini G, Achaz G, Ferretti L. A general framework for neutrality tests based on the site frequency spectrum. Genes (Basel). 2023:14:1714. 10.3390/genes14091714. 37761854 PMC10531300

[evag059-B82] Reuter M, et al Miwi catalysis is required for piRNA amplification-independent LINE1 transposon silencing. Nature. 2011:480:264–267. 10.1038/nature10672. 22121019

[evag059-B83] Robinson MR, et al Population genetic differentiation of height and body mass index across Europe. Nat Genet. 2015:47:1357–1362. 10.1038/ng.3401. 26366552 PMC4984852

[evag059-B84] Rowe HM, et al KAP1 controls endogenous retroviruses in embryonic stem cells. Nature. 2010:463:237–240. 10.1038/nature08674. 20075919

[evag059-B85] Salamun SG, et al The Epstein-Barr Virus BMRF1 protein activates transcription and inhibits the DNA damage response by binding NuRD. J Virol. 2019:93:e01070-19. 10.1128/JVI.01070-19. 31462557 PMC6819917

[evag059-B86] Savaryn JP, et al Human cytomegalovirus pUL29/28 and pUL38 repression of p53-regulated p21CIP1 and Caspase 1 promoters during infection. J Virol. 2013:87:2463–2474. 10.1128/JVI.01926-12. 23236067 PMC3571358

[evag059-B87] Schöpp T, et al TEX15 is an essential executor of MIWI2-directed transposon DNA methylation and silencing. Nat Commun. 2020:11:3739. 10.1038/s41467-020-17372-5. 32719317 PMC7385494

[evag059-B88] Schrader L, Schmitz J. The impact of transposable elements in adaptive evolution. Mol Ecol. 2019:28:1537–1549. 10.1111/mec.14794. 30003608

[evag059-B89] Seczynska M, Lehner PJ. The sound of silence: mechanisms and implications of HUSH complex function. Trends Genet. 2023:39:251–267. 10.1016/j.tig.2022.12.005. 36754727

[evag059-B90] Sherry KP, Das RK, Pappu RV, Barrick D. Control of transcriptional activity by design of charge patterning in the intrinsically disordered RAM region of the Notch receptor. Proc Natl Acad Sci U S A. 2017:114:E9243–E9252. 10.1073/pnas.1706083114. 29078291 PMC5676888

[evag059-B91] Shor B, Schneidman-Duhovny D. CombFold: predicting structures of large protein assemblies using a combinatorial assembly algorithm and AlphaFold2. Nat Methods. 2024:21:477–487. 10.1038/s41592-024-02174-0. 38326495 PMC10927564

[evag059-B92] Simkin A, Wong A, Poh Y-P, Theurkauf WE, Jensen JD. Recurrent and recent selective sweeps in the piRNA pathway: recurrent and recent selective sweeps in the piRNA pathway. Evolution. 2013:67:1081–1090. 10.1111/evo.12011. 23550757 PMC3992950

[evag059-B93] Sironi M, Cagliani R, Forni D, Clerici M. Evolutionary insights into host–pathogen interactions from mammalian sequence data. Nat Rev Genet. 2015:16:224–236. 10.1038/nrg3905. 25783448 PMC7096838

[evag059-B94] Tajima F . Statistical method for testing the neutral mutation hypothesis by DNA polymorphism. Genetics. 1989:123:585–595. 10.1093/genetics/123.3.585. 2513255 PMC1203831

[evag059-B95] Takemoto N, Yoshimura T, Miyazaki S, Tashiro F, Miyazaki J-i. Gtsf1l and Gtsf2 are specifically expressed in gonocytes and spermatids but are not essential for spermatogenesis. PLoS One. 2016:11:e0150390. 10.1371/journal.pone.0150390. 26930067 PMC4773171

[evag059-B96] Tesei G, et al Conformational ensembles of the human intrinsically disordered proteome. Nature. 2024:626:897–904. 10.1038/s41586-023-07004-5. 38297118

[evag059-B97] Tesei G, Lindorff-Larsen K. Improved predictions of phase behaviour of intrinsically disordered proteins by tuning the interaction range. Open Res Europe. 2022:2:94. 10.12688/openreseurope.14967.2. PMC10450847 37645312

[evag059-B98] Tesei G, Schulze TK, Crehuet R, Lindorff-Larsen K. Accurate model of liquid–liquid phase behavior of intrinsically disordered proteins from optimization of single-chain properties. Proc Natl Acad Sci U S A. 2021:118:e2111696118. 10.1073/pnas.2111696118. 34716273 PMC8612223

[evag059-B99] Thomas J, Pritham EJ. *Helitrons*, the eukaryotic rolling-circle transposable elements. Microbiol Spectr. 2015:3:3.4.03. 10.1128/microbiolspec.MDNA3-0049-2014. 26350323

[evag059-B100] Thomas JH, Schneider S. Coevolution of retroelements and tandem zinc finger genes. Genome Res. 2011:21:1800–1812. 10.1101/gr.121749.111. 21784874 PMC3205565

[evag059-B101] Turchin MC, et al Evidence of widespread selection on standing variation in Europe at height-associated SNPs. Nat Genet. 2012:44:1015–1019. 10.1038/ng.2368. 22902787 PMC3480734

[evag059-B102] Venter JC, et al The sequence of the human genome. Science. 2001:291:1304–1351. 10.1126/science.1058040. 11181995

[evag059-B103] Voight BF, Kudaravalli S, Wen X, Pritchard JK. A map of recent positive selection in the human genome. PLoS Biol. 2006:4:e72. 10.1371/journal.pbio.0040072. 16494531 PMC1382018

[evag059-B104] Wang X, Ramat A, Simonelig M, Liu M-F. Emerging roles and functional mechanisms of PIWI-interacting RNAs. Nat Rev Mol Cell Biol. 2023:24:123–141. 10.1038/s41580-022-00528-0. 36104626

[evag059-B105] Wei C, et al MIWI N-terminal RG motif promotes efficient pachytene piRNA production and spermatogenesis independent of LINE1 transposon silencing. PLoS Genet. 2023:19:e1011031. 10.1371/journal.pgen.1011031. 37956204 PMC10681313

[evag059-B106] Wells JN, Feschotte C. A field guide to eukaryotic transposable elements. Annu Rev Genet. 2020:54:539–561. 10.1146/annurev-genet-040620-022145. 32955944 PMC8293684

[evag059-B107] Wernersson R . RevTrans: multiple alignment of coding DNA from aligned amino acid sequences. Nucleic Acids Res. 2003:31:3537–3539. 10.1093/nar/gkg609. 12824361 PMC169015

[evag059-B108] Werren JH . Selfish genetic elements, genetic conflict, and evolutionary innovation. Proc Natl Acad Sci U S A. 2011:108:10863–10870. 10.1073/pnas.1102343108. 21690392 PMC3131821

[evag059-B109] Wicker T, et al A unified classification system for eukaryotic transposable elements. Nat Rev Genet. 2007:8:973–982. 10.1038/nrg2165. 17984973

[evag059-B110] Wickham H . Ggplot2: elegant graphics for data analysis. Springer; 2016.

[evag059-B111] Wu P-H, et al The evolutionarily conserved piRNA-producing locus pi6 is required for male mouse fertility. Nat Genet. 2020:52:728–739. 10.1038/s41588-020-0657-7. 32601478 PMC7383350

[evag059-B112] Yang F, et al TEX15 associates with MILI and silences transposable elements in male germ cells. Genes Dev. 2020:34:745–750. 10.1101/gad.335489.119. 32381626 PMC7263141

[evag059-B113] Yang F, Eckardt S, Leu NA, McLaughlin KJ, Wang PJ. Mouse TEX15 is essential for DNA double-strand break repair and chromosomal synapsis during male meiosis. J Cell Biol. 2008:180:673–679. 10.1083/jcb.200709057. 18283110 PMC2265566

[evag059-B114] Yang F, Wang PJ. Multiple LINEs of retrotransposon silencing mechanisms in the mammalian germline. Seminars in Cell Dev Biol. 2016:59:118–125. 10.1016/j.semcdb.2016.03.001. PMC5011444 26957474

[evag059-B115] Yang Z . PAML: a program package for phylogenetic analysis by maximum likelihood. Comput Appl Biosci. 1997:13:555–556. 10.1093/bioinformatics/13.5.555. 9367129

[evag059-B116] Yang Z . PAML 4: phylogenetic analysis by maximum likelihood. Mol Biol Evol. 2007:24:1586–1591. 10.1093/molbev/msm088. 17483113

[evag059-B117] Yi M, et al Rapid evolution of piRNA pathway in the teleost fish: implication for an adaptation to transposon diversity. Genome Biol Evol. 2014:6:1393–1407. 10.1093/gbe/evu105. 24846630 PMC4079211

[evag059-B118] Yoshimura T, et al Mouse GTSF 1 is an essential factor for secondary pi RNA biogenesis. EMBO Rep. 2018:19:e42054. 10.15252/embr.201642054. 29437694 PMC5891400

[evag059-B119] Yurkovetskiy L, et al Primate immunodeficiency virus proteins Vpx and Vpr counteract transcriptional repression of proviruses by the HUSH complex. Nat Microbiol. 2018:3:1354–1361. 10.1038/s41564-018-0256-x. 30297740 PMC6258279

[evag059-B120] Zarin T, et al Proteome-wide signatures of function in highly diverged intrinsically disordered regions. eLife. 2019:8:e46883. 10.7554/eLife.46883. 31264965 PMC6634968

[evag059-B121] Zarin T, Tsai CN, Nguyen Ba AN, Moses AM. Selection maintains signaling function of a highly diverged intrinsically disordered region. Proc Natl Acad Sci U S A. 2017:114:E1450–E1459. 10.1073/pnas.1614787114. 28167781 PMC5338452

[evag059-B122] Zhang H, et al The piRNA pathway is essential for generating functional oocytes in golden hamsters. Nat Cell Biol. 2021:23:1013–1022. 10.1038/s41556-021-00750-6. 34489574

[evag059-B123] Zhang Y, Skolnick J. TM-align: a protein structure alignment algorithm based on the TM-score. Nucl Acids Res. 2005:33:2302–2309. 10.1093/nar/gki524. 15849316 PMC1084323

[evag059-B124] Zhao B, et al Intrinsic disorder in human RNA-binding proteins. J Mol Biol. 2021:433:167229. 10.1016/j.jmb.2021.167229. 34487791

[evag059-B125] Zhao S, et al TNRC18 engages H3K9me3 to mediate silencing of endogenous retrotransposons. Nature. 2023:623:633–642. 10.1038/s41586-023-06688-z. 37938770 PMC11000523

[evag059-B126] Zheng W, Dignon G, Brown M, Kim YC, Mittal J. 2020. Hydropathy patterning complements charge patterning to describe conformational preferences of disordered proteins. J Phys Chem Lett. 11(9):3408–3415. 10.1021/acs.jpclett.0c00288. 32227994 PMC7450210

