# Supplementary material for: Positive Selection Targeted Primate Genes that Encode Transposable Element Repressors
Source: Genome Biol Evol. 2026 Mar 5;18(3):evag059. doi: 10.1093/gbe/evag059 (PMC12994711; doi:10.1093/gbe/evag059)

Figure S1. Structural model of the human PIWIL2-GTSF1-MAEL-piRNA-target duplex-TDRD9 complex (prediction score = 0.758).

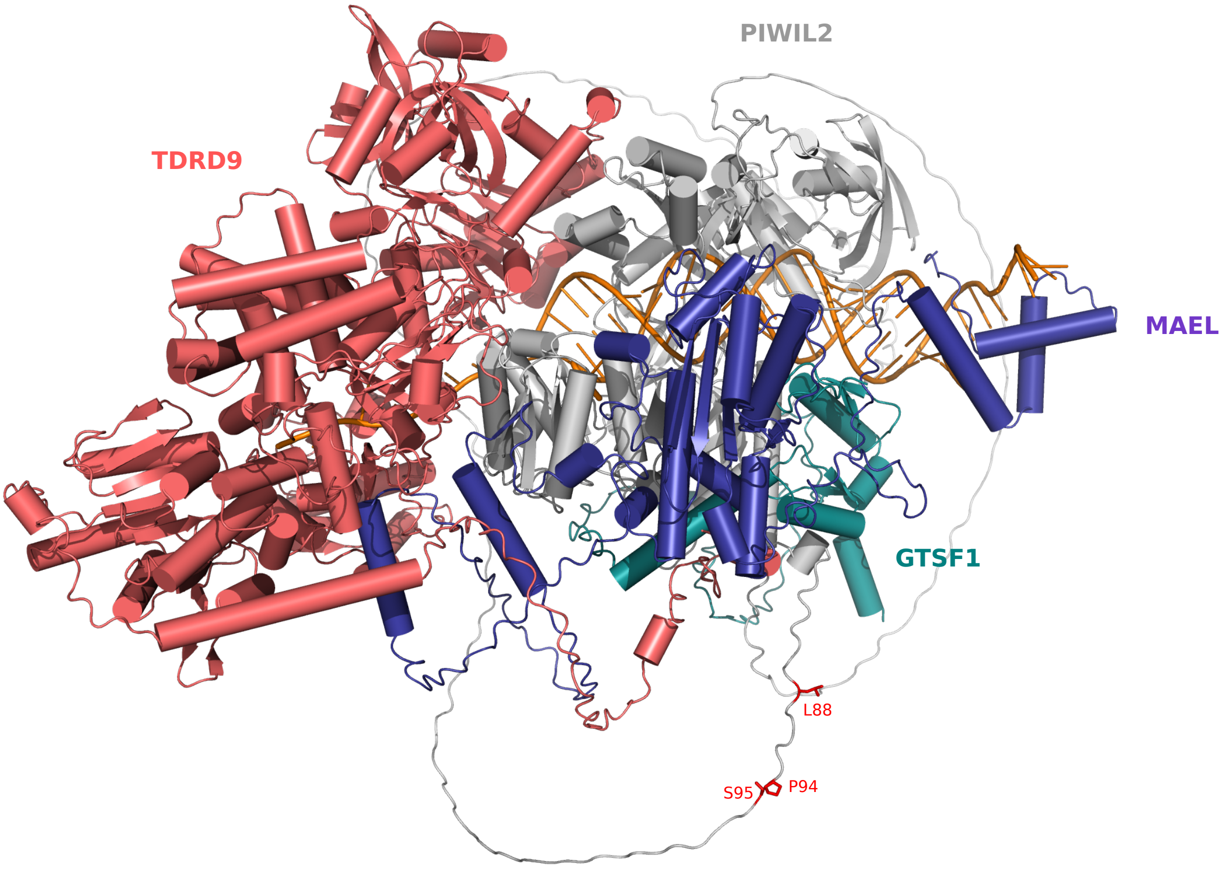

Supplement: evag059_Supplementary_Data [file evag059_supplementary_data.zip › FigureS1.pdf]
