# Supplementary material for: Positive Selection Targeted Primate Genes that Encode Transposable Element Repressors
Source: Genome Biol Evol. 2026 Mar 5;18(3):evag059. doi: 10.1093/gbe/evag059 (PMC12994711; doi:10.1093/gbe/evag059)

Figure S2. Intrinsically disorder regions in TE control genes. Comparison of the fraction of disorder estimated by Metapredict or annotated in the MobiDB database for the sixty TE control genes.

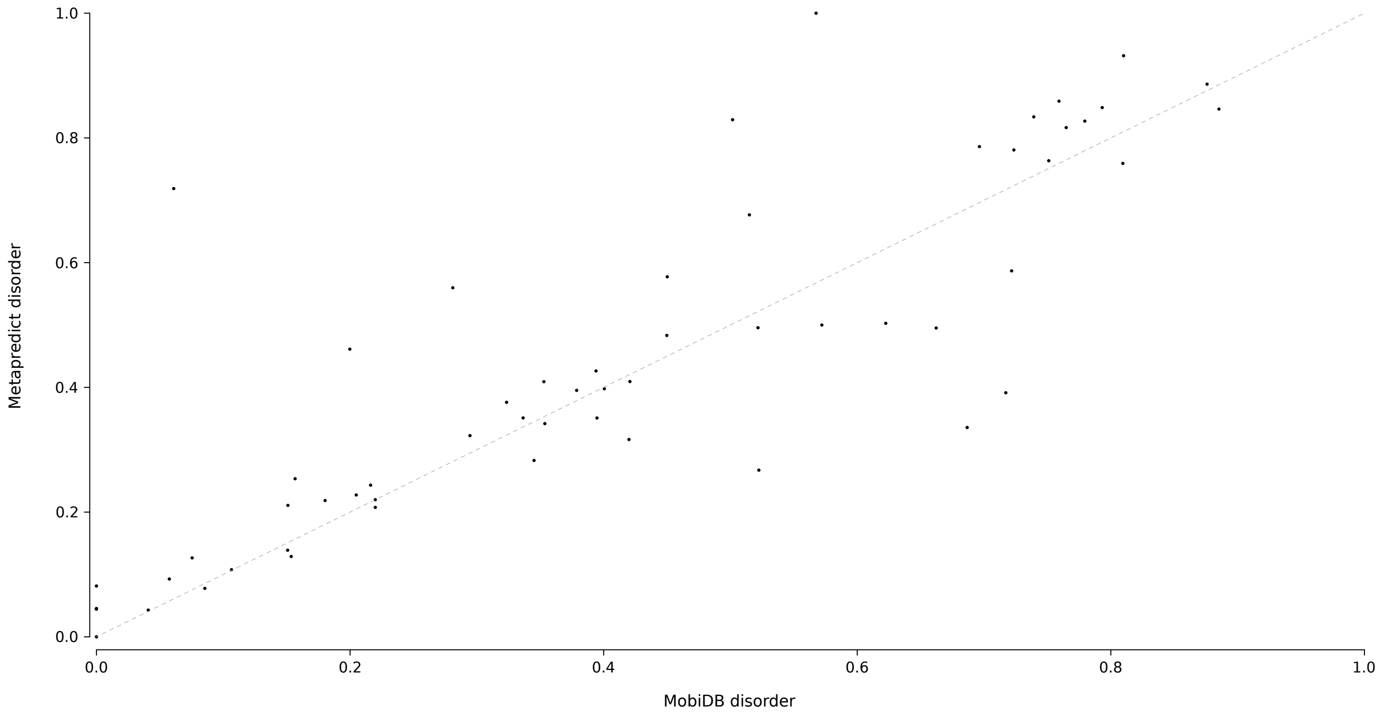

Supplement: evag059_Supplementary_Data [file evag059_supplementary_data.zip › FigureS2.pdf]
